# Mass Antibiotic Treatment for Group A Streptococcus Outbreaks in Two Long-Term Care Facilities^1^

**DOI:** 10.3201/eid0910.030130

**Published:** 2003-10

**Authors:** Andrea Smith, Aimin Li, Ornella Tolomeo, Gregory J. Tyrell, Frances Jamieson, David Fisman

**Affiliations:** * City of Hamilton Social and Public Health Services Department, Hamilton, Ontario, Canada; † University of Alberta, Edmonton, Alberta; ‡ Canada Ontario Ministry of Health and Long-Term Care, Toronto, Ontario, Canada; § Provincial Laboratory for Public Health, Edmonton, Alberta, Canada; ¶ McMaster University, Hamilton, Ontario, Canada;

## Abstract

Outbreaks of invasive infections caused by group A β-hemolytic streptococcus (GAS) may occur in long-term care settings and are associated with a high case-fatality rate in debilitated adults. Targeted antibiotic treatment only to residents and staff known to be at specific risk of GAS may be an ineffective outbreak control measure. We describe two institutional outbreaks in which mass antibiotic treatment was used as a control measure. In the first instance, mass treatment was used after targeted antibiotic treatment was not successful. In the second instance, mass treatment was used to control a rapidly evolving outbreak with a high case-fatality rate. Although no further clinical cases were seen after the introduction of mass antibiotic treatment, persistence of the outbreak strain was documented in one institution >1 year after cases had ceased. Strain persistence was associated with the presence of a chronically colonized resident and poor infection control practices.

Group A β-hemolytic streptococcus (GAS) has a longstanding association with pharyngitis, skin and soft tissue infection, and pneumonia (*1*–*3*). In the past decade, reports of GAS-associated necrotizing fasciitis and streptococcal toxic shock syndrome (TSS) have increased (*2*,*4*). Increasingly, outbreaks of invasive GAS are recognized in long-term care facilities (*5*–*10*). In the Canadian province of Ontario, 4% of invasive GAS cases occur in long-term care facilities, and of these cases, one third are outbreak associated (*5*). In long-term care settings, a high case-fatality rate has been observed (40% to 60%) (*5**,**11**,**12*).

Guidelines for control of GAS outbreaks in nursing homes were developed by the Ontario Ministry of Health in 1995 (*13*). These guidelines emphasize the identification and elimination of colonization through providing antibiotics to those known to be carriers of the bacterium. Although such an approach should minimize antibiotic exposure, carrying it out in practice could be difficult, as cultures may be falsely negative (*14*), and persons could become colonized or transmit colonization to others in the interval between culturing and treatment. Antibiotic treatment of all residents and staff would not be subject to such limitations. We present two outbreaks of invasive GAS infection in long-term care facilities in Hamilton, Ontario, Canada. Each was caused by transmission of a single well-characterized outbreak strain, and in both cases, mass antibiotic treatment was used to control the outbreak.

## Methods

### Epidemiologic Investigations

In the province of Ontario, invasive infections with GAS (isolation of GAS from blood or an otherwise sterile site) and cases of suspected TSS and necrotizing fasciitis must be reported to local public health authorities (*15*). Reports of invasive GAS infection originating from long-term care institutions in the city of Hamilton resulted in an epidemiologic investigation by the City of Hamilton Social and Public Health Services Department. In the two outbreaks described below, investigations consisted of site visits with line-listings created to identify facility residents and staff; reviews of recent admissions, discharges, and deaths among residents; and reviews of reports of illness among residents and staff. Environmental investigations and reviews of infection control practices were also performed.

Attempts were made to identify facility residents and staff with close epidemiologic linkage to the index patients (e.g., caregivers, roommates, and close friends). Although typically only persons with epidemiologic linkage to index patients are cultured for possible GAS carriage, the circumstances described below resulted in more widespread collection of culture specimens. Specimens were obtained from noses and pharynges of residents and staff, and attempts were made to obtain cultures of perirectal areas and wounds in facility residents. In the second outbreak described below, staff provided self-collected rectal and vaginal swabs.

### Microbiologic Evaluation and Characterization of GAS Strains

Swabs from institution residents and staff were plated onto blood agar, with group A streptococcus identified by β-hemolysis and Lancefield typing using standard commercially available latex agglutination methods, as described elsewhere (*16*). Isolates were subsequently evaluated for relatedness by using pulsed-field gel electrophoresis (PFGE). Briefly, PFGE was performed using fresh overnight cultures of GAS strains. Isolates were suspended in buffered saline, and solutions were adjusted in volume until optical densities (OD) were identical (1.6 OD at 610 nm). To prepare genomic DNA, 500 μL of cell suspension from each strain was mixed with an equal volume of 2% low melting agarose solution. The agarose plug was prepared by using a commercially available plug mould (Bio-Rad Canada Limited, Mississauga, Ontario, Canada). All GAS plugs were treated in lysis buffer containing 50 μg/mL lysozyme at 37°C overnight, extensively washed in washing buffer, and then treated in a solution containing 50 μg/mL protease K at 55°C overnight. All plugs were extensively washed before being restricted by Sma-I enzyme. PFGE was run by using a 1% agarose gel at 6 volts/cm; run time was 26 h with an initial switch time of 20 s and a final switch time of 60 s. Following gel electrophoresis, the gel was stained in a solution containing 0.5 μg/mL ethidium bromide and photographed by using the Bio-Rad Multi-analysis CCD camera system. ATCC strain 19615 was included as the quality control strain.

M and T serologic typing and opacity factor determination were performed on all isolates with standardized methods (*17*). In addition, antiopacity factor (AOF) typing was performed on all isolates that were opacity factor–positive (*17*). All antisera were prepared in-house. Because of the difficulty of producing antisera for some M types, AOF typing has been frequently used to predict the M type (*18*,*19*). Although AOF typing does not always have the same type specificity as M typing, this approach is considered valid for most strains from developed countries (*20*). AOF typing was used to predict the following M types: 9, 11, 25, 28, 48, 77, 78 and 92 (PT5118).

## Results

### Outbreak 1

In October 2000, the Hamilton public health unit was notified of a 97-year-old female nursing home resident (index case-patient 1) admitted to an area hospital with cellulitis and group A streptococcal bacteremia. The nursing home was a 386-bed facility in a suburban area. Cultures were obtained as described from 87 residents and staff who might have had contact with the person with invasive disease. One resident had a throat culture positive for group A streptococcus. This isolate was genetically indistinguishable by PFGE analysis from the invasive isolate obtained from index case-patient 1, and both strains were typed as M77 T9/10, and positive for serum opacity factor.

Intervention consisted of reinforcement of standard infection control practices with facility staff and treatment of the person with pharyngeal colonization with a 10-day course of cephalexin, 250 mg four times per day. Follow-up cultures from this person taken 1 month after completion of treatment were negative. Index case-patient 1 died during the course of the investigation of apparently unrelated cardiac disease.

In January 2001, an 87-year-old female resident of the same long-term care facility was transported to hospital with fever (index case-patient 2), and GAS was isolated from blood cultures drawn at admission. Several days later, GAS was isolated from the wound of another facility resident. Screening cultures performed on 195 residents and staff with a history of contact with one of these two infected persons indicated that seven residents and one staff member were colonized with GAS. PFGE showed that the strains from all resident carriers were identical to the wound and blood isolates, and to the isolate obtained from index case 1. The bacterial isolate obtained from the staff member was determined by PFGE to be unrelated to the outbreak strain. Colonized residents and staff were treated with cephalexin as had been done with the previous patient. Cultures obtained from treated persons 1 month after completion of therapy were again negative.

**Figure 1:**
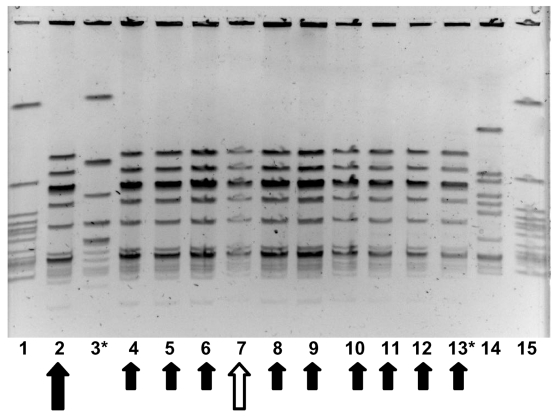
Molecular epidemiology of group A streptococcus (GAS) strains in outbreak. Pulsed field gel electrophoresis, demonstrating relatedness of group A streptococcal isolates from an person with clinical illness from GAS, a person with chronic colonization with GAS, and asymptomatically colonized facility staff and residents. Lanes 1 and 15 contain an ATCC quality control strain. Lane 14 contains an isolate from another nursing facility, unrelated to outbreak 1. The isolate in lane 2 (large solid arrow) was obtained from index case-patient 2 in January 2001. The isolate in lane 7 (large hollow arrow) was obtained from a person with chronic GAS colonization (resident A) in May 2001. Small solid arrows denote electrophoretically identical GAS strains from other persons with asymptomatic colonization with group A streptococcus in May 2001. Asterisk denotes staff member.

In May 2001, another resident of this nursing home was transported to hospital with GAS bacteremia (index case-patient 3). At this time, all 386 residents and 135 staff of the facility were screened for GAS carriage by culturing nasopharynx specimens. Eleven residents and three staff members were identified as carrying group A streptococcus. Thirteen of these (11 residents and two staff members) subsequently proved to be carrying strains identified by PFGE analysis to be identical to the strain obtained from index case-patient 2 (Figure 1) (prevalence of colonization or infection among residents was 2.8% vs. prevalence of colonization 1.5% among staff , p=0.38 by chi-square test).

One of the residents colonized with GAS (resident A) was a 69-year-old woman, immobilized by severe neurologic disease, who had a suprapubic bladder catheter and a gastrostomy tube. Resident A was subsequently determined to have had urine and wound cultures positive for GAS in July 2000, 3 months before index case-patient 1 sought treatment; these isolates were unavailable for evaluation. After consultation with local communicable disease control experts, the public health team recommended administering antibiotics to all facility residents and staff. All residents were treated with a 10-day course of cephalexin or penicillin VK, with erythromycin given to persons allergic to β-lactam agents. Similar antibiotic regimens were recommended for all staff members. No data are available with regard to the degree of compliance with antibiotic therapy among staff. However, all previously colonized staff members were culture-negative for group A streptococcus on repeat culturing 1 month later.

Ten of 11 treated residents had cultures negative for group A streptococcus 1 month after treatment. Resident A remained persistently colonized at gastrostomy and catheter sites, despite a subsequent prolonged course of oral clindamycin. This resident was placed on contact isolation precautions, with staff using gowns and gloves when participating in her care. She died in July 2002.

**Figure 2:**
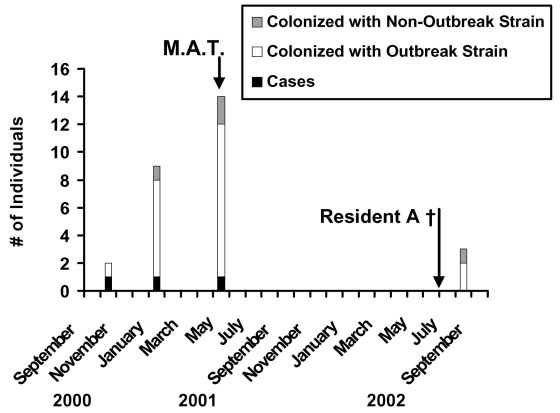
Epidemic curve for outbreak 1. Clinical cases (black bars) of invasive GAS infection occurred at intervals of 3 to 4 months. With the occurrence of cases, acquisition of culture specimens resulted in identification of asymptomatic colonization with the outbreak strain (white bars) or unrelated strains (hatched bars) in other residents and staff. No additional clinical cases occurred after mass antibiotic treatment (M.A.T.); resident A died (†) in July 2002; colonization of two residents with the outbreak strain was recognized 1 month later.

No subsequent cases of invasive group A streptococcal disease have been reported at this facility as of February 2003. However, in August 2002, GAS was identified in cultures of eye drainage and wound drainage from three residents of a single wing of the facility (Figure 2). PFGE demonstrated two of the three isolates to be indistinguishable by PFGE from isolates obtained from resident A. Neither of these persons had been present in the facility at the time of invasive cases, and neither had been housed in the same part of the facility as resident A. An audit of infection control procedures at that time showed several violations of standard infection control measures, including the reuse of a single washcloth on multiple residents of the facility.

### Outbreak 2

In November 2001, the Hamilton public health unit was notified of two deaths from invasive group A streptococcal disease at a 126-bed long-term care facility. The first case-patient was a 78-year-old man who had been admitted to the hospital with fever and possible pneumonia 2 days before. A blood culture grew GAS. The second case-patient was a 61-year-old man with Parkinson’s disease and dementia who had been admitted to the hospital 1 day before with soft skin tissue infection and likely necrotizing fasciitis. GAS was subsequently isolated from blood cultures. Both patients died on the day the cases were reported to the public health department.

A site visit was performed. The facility had 127 residents and 150 staff members. The two cases had originated on different floors of the facility (2nd and 4th). A large holiday party had been held in the communal dining hall 4 days previously, providing an opportunity for residents, staff, and community members to mingle. In addition, a healthcare worker reportedly had pharyngitis, a rash, and desquamation of the palms and had received a diagnosis of “scarlet fever” 3 weeks earlier.

**Figure 3:**
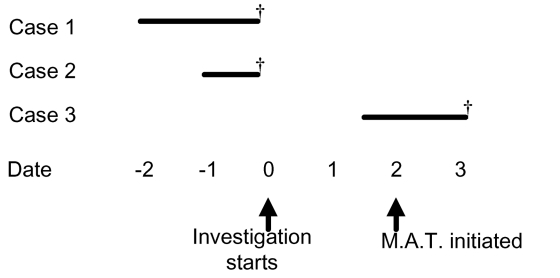
Timeline for outbreak 2. Solid lines represent the time of onset and duration of illness among three cases with invasive GAS infection in outbreak 2, relative to the initiation of the outbreak investigation (date=0). Daggers (†) denote death. Mass antibiotic treatment was started 2 days after the investigation was initiated.

Due to the lack of any epidemiologic link between case-patients and the population mixing that had occurred at the holiday party, swab cultures were collected on all facility residents and all available staff members. Epidemic control measures were introduced and included restriction of new admissions to the facility and reinforcement of infection control practices. Based on the rapidity of events, the apparent high case-fatality rate, and recent experience with the failure of targeted culturing and treatment (as described in outbreak 1), a decision was made to initiate mass antibiotic treatment for all facility residents and staff. After that decision, but before mass antibiotic treatment could be initiated, an 89-year-old male resident was admitted to the hospital with “congestive heart failure.” Gram stain of sputum indicated gram-positive cocci in chains. This man died 36 h after admission; GAS was isolated from blood cultures taken at hospital admission (Figure 3).

Mass antibiotic treatment was initiated approximately 48 h after the start of the investigation, with azithromycin, 250 mg orally each day, administered for 5 days to residents and to staff who wished to be treated at the facility. Staff who wished to receive antibiotic treatment from their personal physicians were permitted to do so. Subsequently, GAS was isolated from nasal and pharyngeal cultures of two staff members and five residents of the facility. GAS was also isolated from a wound culture from a facility resident.

All colonized residents were negative for GAS on repeat cultures, 4 weeks after receiving antibiotics. One staff member remained culture positive 2 weeks after completing a course of cephalexin provided by her personal physician; she received two additional courses of β-lactam antibiotics and was documented to be culture negative for GAS 4 weeks after she completed the second antibiotic course.

**Figure 4:**
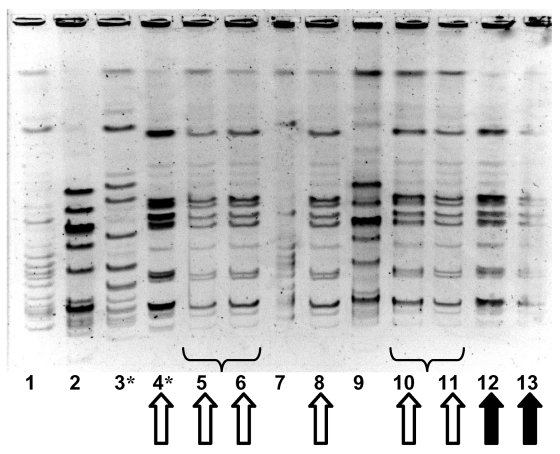
Molecular epidemiology of group A streptococcal strains in outbreak 2. Pulsed-field gel electrophoresis, demonstrating relatedness of group A streptococcal isolates from facility staff and residents. Lanes 1 and 7 contain an ATCC quality control strain. Solid arrows denote identical strains from two of the three persons in whom fatal invasive group A streptococcal infection developed; the third person with invasive disease had an electrophoretically identical strain (not shown). Hollow arrows denote identical strains from persons with asymptomatic colonization with group A streptococcus. Brackets denote duplicate strains from the same person; asterisk denotes staff member.

PFGE performed on the three GAS strains from case-patients and the seven strains from colonized residents and staff showed that four colonized persons had isolates identical to those from the deceased case-patients; all were serotype M1 T1 and were serum opacity factor negative (Figure 4). The remaining three isolates represented two distinct strains unrelated to the outbreak strain. The proportion of residents colonized or infected with the outbreak strain was significantly higher than the proportion of staff colonized (4.7% vs. 0.7%, p=0.03 by chi-square test).

## Discussion

Our description of outbreaks of multiple cases of invasive disease attributable to a single, identified strain of GAS in long-term care institutions is consistent with prior reports (*5*–*10*). Outbreak 1 was caused by an M77 strain, an uncommon cause of invasive streptococcal disease in Canada (*21*). The second outbreak was caused by an M1-type strain, the most common isolate type in Canada, which may be more virulent than other serotypes (*21*,*22*).

Epidemiologic investigation of these two outbreaks showed a higher prevalence of colonization with the outbreak strains among residents than among staff, although this difference was not statistically significant in outbreak 1. Given the limited mobility of the residents of both facilities, such a difference would be consistent with disease transmission involving asymptomatically colonized healthcare workers. Transmission may also have occurred as a result of transient carriage of the organism by healthcare workers, as may occur with nosocomial transmission of *Clostridium difficile* and vancomycin-resistant enterococcus (*23*,*24*), or as a result of contaminated fomites, such the washcloth described in outbreak 1. Other possible mechanisms of transmission include direct resident-to-resident transmission in the context of such social events as the holiday party described in outbreak 2 and foodborne transmission (*25*).

Any of these mechanisms of spread could limit the expected effectiveness of screening and targeted antibiotic treatment, as culturing of only residents and staff with a clear epidemiologic link to cases would not be expected to identify all colonized persons. This situation was well demonstrated in outbreak 1, which involved probable worker transmission of a strain from an immobile, chronically colonized person (resident A) to other residents in this institution. Although likely important in the perpetuation of the outbreak, resident A was identified incidentally when cultures were performed on all facility residents 8 months after the initial case of invasive disease was reported. The importance of silent carriage of GAS in the perpetuation of outbreaks has been well-described in both healthcare (*26*,*27*) and community (*28*) contexts.

Our use of mass antibiotic treatment as an epidemic control measure appears to have been successful, inasmuch as no further cases of invasive GAS disease have been reported from either facility at the time of writing (February 2003). However, the documented persistence of the bacterial strain implicated in outbreak 1 after the death of resident A suggests that breaches in infection control practices (as identified during site visits), combined with the presence of a persistently colonized resident or staff person, may limit the effectiveness of this control measure. Such a control measure would also not prevent the repeated reimportation of a circulating community strain of GAS into a facility by healthcare workers or visiting family members (*29*,*30*).

The impact of wide-scale antibiotic use on pathogenic microorganisms in long-term care institutions also remains an issue of concern. Resistance to macrolides among GAS isolates appears to be increasing in frequency (*31*,*32*), and the overuse of β-lactam antibiotics and macrolides may adversely affect the antibiotic susceptibility patterns of such common pathogens as *Streptococcus pneumoniae* (*33*–*36*).

These two outbreaks of invasive GAS in the long-term care setting highlight the limitations of targeted culturing and antibiotic treatment as an outbreak control strategy. However, our experience also highlights the limitations of mass treatment as a control strategy, particularly when poor infection control practices persist and chronically colonized residents are present. Further research into the epidemiology and transmission of GAS in the long-term care setting will help refine the optimal approach to managing these challenging outbreaks.

## References

[1] Kiselica D. Group A beta-hemolytic streptococcal pharyngitis: current clinical concepts. Am Fam Physician. 1994;49:1147–54. 8154403

[2] Bisno AL, Stevens DL. Streptococcal infections of skin and soft tissues. N Engl J Med. 1996;334:240–5. 10.1056/NEJM199601253340407 8532002

[3] Barnham M, Weightman N, Anderson A, Pagan F, Chapman S. Review of 17 cases of pneumonia caused by *Streptococcus pyogenes.* Eur J Clin Microbiol Infect Dis. 1999;18:506–9. 10.1007/s100960050333 10482030 PMC7102171

[4] Baracco GJ, Bisno AL. Therapeutic approaches to streptococcal toxic shock syndrome. Curr Infect Dis Rep. 1999;1:230–7. 10.1007/s11908-999-0024-4 11095793

[5] Davies HD, McGeer A, Schwartz B, Green K, Cann D, Simor AE, Invasive group A streptococcal infections in Ontario, Canada. Ontario Group A Streptococcal Study Group. N Engl J Med. 1996;335:547–54. 10.1056/NEJM199608223350803 8684408

[6] Centers for Disease Control and Prevention. Nursing home outbreaks of invasive group A streptococcal infections—Illinois, Kansas, North Carolina, and Texas. MMWR Morb Mortal Wkly Rep. 1990;39:577–9. 2117240

[7] Auerbach S, Schwartz B, Williams D, Fiorilli MG, Adimora AA, Breiman RF, Outbreak of invasive group A streptococcal infections in a nursing home. Arch Intern Med. 1992;152:1017–22. 10.1001/archinte.152.5.1017 1580705

[8] Harkness GA, Bentley DW, Mottley M, Lee J. *Streptococcus pyogenes* outbreak in a long-term care facility. Am J Infect Control. 1992;20:142–8. 10.1016/S0196-6553(05)80181-6 1636935

[9] Ruben FL, Norden CW, Heisler B, Korica Y. An outbreak of *Streptococcus pyogenes* infections in a nursing home. Ann Intern Med. 1984;101:494–6. 6383164 10.7326/0003-4819-101-4-494

[10] Schwartz B, Elliott JA, Butler JC, Simon PA, Jameson BL, Welch E, Clusters of invasive group A streptococcal infections in family, hospital, and nursing home settings. Clin Infect Dis. 1992;15:277–84. 1520763 10.1093/clinids/15.2.277

[11] Kaul R, McGeer A, Low DE, Green K, Schwartz B. Population-based surveillance for group A streptococcal necrotizing fasciitis: clinical features, prognostic indicators, and microbiologic analysis of seventy-seven cases. Am J Med. 1997;103:18–24. 10.1016/S0002-9343(97)00160-5 9236481

[12] Bucher A, Martin PR, Hoiby EA, Halstensen A, Odegaard A, Helkum KB, Spectrum of disease in bacteraemic patients during a *Streptococcus pyogenes* serotype M-1 epidemic in Norway in 1988. Eur J Clin Microbiol Infect Dis. 1992;11:416–26. 10.1007/BF01961856 1425712

[13] Guidelines for management of contacts of cases of invasive group A streptococcal disease (GAS) including streptococcal toxic shock syndrome (STSS) and necrotizing fasciitis. 1995. Toronto, Ontario Ministry of Health. [Accessed January 28, 2003] Available from: URL: http://microbiology.mtsinai.on.ca/protocols/pdf/k5b.pdf

[14] Pichichero ME. Culture and antigen detection tests for streptococcal tonsillopharyngitis. Am Fam Physician. 1992;45:199–205. 1728090

[15] Health Protection and Promotion Act. Ontario Regulation 559/91, R22.1. 4-23-1999.

[16] Koneman E, Allen S, Janda W, Schreckenberger PC, Winn WC. The gram-positive cocci part II: streptococci, enterococci, and “streptococcus-like bacteria.” Color atlas and textbook of diagnostic microbiology, 5th ed. Philadelphia: Lippincott; 1997. p. 577–649.

[17] Johnson DR, Sramek J, Kaplan EI, Bicova R, Havlicek J, Havlickova H, Laboratory diagnosis of group A streptococcal infections. Geneva: World Health Organization; 1996.

[18] Facklam R, Beall B, Efstratiou A, Fischetti V, Johnson D, Kaplan E, *emm* typing and validation of provisional M types for group A streptococci. Emerg Infect Dis. 1999;5:247–53. 10.3201/eid0502.990209 10221877 PMC2640698

[19] Maxted W, Widdowson J, Fraser C. The use of the serum opacity reaction in the typing of group A streptococci. J Med Microbiol. 1973;6:83–90. 10.1099/00222615-6-1-83 4734813

[20] Beall B, Gherardi G, Lovgren M, Facklam RR, Forwick BA, Tyrrell GJ. *emm* and *sof* gene sequence variation in relation to serological typing of opacity-factor-positive group A streptococci. Microbiology. 2000;146:1195–209. 10832648 10.1099/00221287-146-5-1195

[21] Tyrrell GJ, Lovgren M, Forwick B, Hoe NP, Musser JM, Talbot JA. M types of group a streptococcal isolates submitted to the National Centre for *Streptococcus* (Canada) from 1993 to 1999. J Clin Microbiol. 2002;40:4466–71. 10.1128/JCM.40.12.4466-4471.2002 12454137 PMC154642

[22] Talkington DF, Schwartz B, Black CM, Todd JK, Elliott J, Breiman RF, Association of phenotypic and genotypic characteristics of invasive *Streptococcus pyogenes* isolates with clinical components of streptococcal toxic shock syndrome. Infect Immun. 1993;61:3369–74. 8335368 10.1128/iai.61.8.3369-3374.1993 PMC281012

[23] Samore MH. Epidemiology of nosocomial *Clostridium difficile* diarrhoea. J Hosp Infect. 1999;43(Suppl):S183–90. 10.1016/S0195-6701(99)90085-3 10658778

[24] Austin DJ, Bonten MJ, Weinstein RA, Slaughter S, Anderson RM. Vancomycin-resistant enterococci in intensive-care hospital settings: transmission dynamics, persistence, and the impact of infection control programs. Proc Natl Acad Sci U S A. 1999;96:6908–13. 10.1073/pnas.96.12.6908 10359812 PMC22015

[25] Farley TA, Wilson SA, Mahoney F, Kelso KY, Johnson DR, Kaplan EL. Direct inoculation of food as the cause of an outbreak of group A streptococcal pharyngitis. J Infect Dis. 1993;167:1232–5. 8486961 10.1093/infdis/167.5.1232

[26] Mastro T, Farley T, Elliott J, Facklam RR, Perks JR, Hadler JL, An outbreak of surgical-wound infections due to group A streptococcus carried on the scalp. N Engl J Med. 1990;323:968–72. 10.1056/NEJM199010043231406 2205801

[27] Paul S, Genese C, Spitalny K. Postoperative group a beta-hemolytic *Streptococcus* outbreak with the pathogen traced to a member of a healthcare worker's household. Infect Control Hosp Epidemiol. 1990;11:643–6. 10.1086/646115 2273228

[28] Cockerill FR III, MacDonald KL, Thompson RL, Robertson F, Kohner PC, Besser-Wiek J, An outbreak of invasive group A streptococcal disease associated with high carriage rates of the invasive clone among school-aged children. JAMA. 1997;277:38–43. 10.1001/jama.277.1.38 8980208

[29] Weber DJ, Rutala WA, Denny FW Jr. Management of healthcare workers with pharyngitis or suspected streptococcal infections. Infect Control Hosp Epidemiol. 1996;17:753–61. 10.1086/647223 8934245

[30] Kaplan EL, Wotton JT, Johnson DR. Dynamic epidemiology of group A streptococcal serotypes associated with pharyngitis. Lancet. 2001;358:1334–7. 10.1016/S0140-6736(01)06415-7 11684215

[31] Martin JM, Green M, Barbadora KA, Wald ER. Erythromycin-resistant group A streptococci in schoolchildren in Pittsburgh. N Engl J Med. 2002;346:1200–6. 10.1056/NEJMoa013169 11961148

[32] Weiss K, de Azavedo J, Restieri C, Galarneau LA, Gourdeau M, Harvey P, Phenotypic and genotypic characterization of macrolide-resistant group A Streptococcus strains in the province of Quebec, Canada. J Antimicrob Chemother. 2001;47:345–8. 10.1093/jac/47.3.345 11222568

[33] Fry AM, Jha HC, Lietman TM, Chaudhary JS, Bhatta RC, Elliott J, Adverse and beneficial secondary effects of mass treatment with azithromycin to eliminate blindness due to trachoma in Nepal. Clin Infect Dis. 2002;35:395–402. 10.1086/341414 12145722

[34] Granizo JJ, Aguilar L, Casal J, Dal-Re R, Baquero F. *Streptococcus pneumoniae* resistance to erythromycin and penicillin in relation to macrolide and beta-lactam consumption in Spain (1979–1997). J Antimicrob Chemother. 2000;46:767–73. 10.1093/jac/46.5.767 11062196

[35] Kristinsson KG. Effect of antimicrobial use and other risk factors on antimicrobial resistance in pneumococci. Microb Drug Resist. 1997;3:117–23. 10.1089/mdr.1997.3.117 9185137

[36] Morita JY, Kahn E, Thompson T, Laclaire L, Beall B, Gheradi G, Impact of azithromycin on oropharyngeal carriage of group A Streptococcus and nasopharyngeal carriage of macrolide-resistant *Streptococcus pneumoniae.* Pediatr Infect Dis J. 2000;19:41–6. 10.1097/00006454-200001000-00009 10643849

